# Evaluation of Bacterial Profiles and Antibiotic Susceptibility in Orthopedic Patients With Surgical Site Infections After Implant Surgery

**DOI:** 10.7759/cureus.83305

**Published:** 2025-05-01

**Authors:** Shah Waliullah, Utkarsh Upadhyay, Asma Khan, Sheetal Verma, Mayank Mahendra, Shailendra Singh, Zeenat Ara

**Affiliations:** 1 Department of Orthopedic Surgery, King George's Medical University, Lucknow, IND; 2 Department of Microbiology, King George's Medical University, Lucknow, IND; 3 Department of Orthopedics, King George's Medical University, Lucknow, IND

**Keywords:** erythema, infectious agent, prospective study, s. aureus, surgical site infection (ssi)

## Abstract

Introduction: Surgical site infections (SSIs) are a significant concern for surgeons and a leading cause of poor patient outcomes, increased morbidity, extended hospital stay, and higher costs. SSIs are of particular concern in orthopedic implant surgeries because biofilms on implants protect bacteria. This study aimed to evaluate the incidence rate of SSIs in orthopedic implant patients, identify associated risk factors, and analyze the types of bacteria causing these infections.

Methods and materials: This study was a prospective analysis of 1,282 patients who underwent orthopedic implant surgery at the Orthopedic Department over a period of more than one year. It examined various parameters, including the type of surgery, surgical site, associated risk factors, and duration of surgery. It also explored the correlation between the development of SSIs, the time to development, and the associated risk factors.

Results: Of the 1282 orthopedic patients followed up, 98 developed SSI. Thus, the incidence rate was 7.64%. Patients undergoing emergency surgery are more likely to develop infections than those undergoing elective surgery. About 65% of all SSIs were caused by the most prevalent infectious agent, *Staphylococcus aureus*, which infected 23 subjects (22.54%), followed by *Escherichia coli* in 16 subjects (15.68%), *Klebsiella pneumoniae* in 14 subjects (13.72%), and *Acinetobacter baumannii* in 13 subjects (12.74%). The most important risk factor was diabetes, which affected 26 participants with the highest prevalence (26.5%), followed by 16 subjects who used tobacco (16.3%). A further significant risk factor for higher rates of SSIs was longer operation times.

Conclusion: Our analysis highlights the urgent need for a comprehensive antibiotic policy for patients undergoing implant surgery, given the prevalence of common bacteria and the significant risk factors associated with SSIs. To address this critical issue, we propose a level 1 multicenter study to develop standardized guidelines for antibiotic administration in these patients. This approach ensures optimal treatment outcomes and enhances patient safety.

## Introduction

Surgical site infections (SSIs) significantly impact a surgeon's success. They can undermine the surgeon's pride and sense of achievement, leading to serious complications that negatively affect the surgical outcomes. SSIs often result in increased morbidity, longer hospital stays, higher medical costs, and decreased trust between patients and surgeons [1].

SSIs are characterized by symptoms such as pain, redness (erythema), swelling, and discharge from the wound site. Early SSIs occur within the first 30 days following surgery, whereas late SSIs develop more than 30 days after surgery or after a prosthetic implant. Late SSIs can sometimes appear months or even years later [2]. The incidence of SSI has been observed to be significantly higher in developing countries owing to a lack of better postoperative management. Various studies have shown that the worldwide prevalence of SSI in orthopedic implant surgery ranges from 0.7% to 4.3% [3-8], and the incidence is estimated between 1% and 10% [9-12].

Considering the pervasive nature of SSIs, a thorough investigation of the underlying causes and contributing risk factors is essential. This study aimed to meticulously evaluate the incidence of SSIs among patients undergoing orthopedic implant procedures. Additionally, it seeks to identify the various risk factors associated with these infections and analyze the specific types of bacteria implicated in their onset.

## Materials and methods

This prospective study was conducted between September 2023 and August 2024. The study was conducted in the Department of Orthopedic Surgery and the Department of Microbiology at King George's Medical University, Lucknow, India. This study was approved by the Institutional Ethics Committee of King George's Medical University, Lucknow, India (reference code: XVI-PGTSC-IIA/P51). Subjects with SSIs following implant surgery were selected based on the specific inclusion and exclusion criteria. The study was conducted for 12 months.

Sample size

The sample size is determined by using the following formula [13]: N = (Zα / 2) * 2P (1 - P) / d * 2, where N represents the sample size, P represents the prevalence, α represents the error, d represents the degree of freedom, and Zα/2 represents the differentiation coefficient (1.96 or 2) here based on the findings by Mathur et al. [14]. The prevalence of the study was reported as 6.7% (p = 0.067). Thus, substituting these values in the above formula, the calculated sample was approximately 100 cases.

Inclusion criteria

The inclusion criteria for enrolling subjects were as follows: age between 18 and 75 years, undergoing elective orthopedic surgeries or emergency simple fracture fixation, a confirmed diagnosis of infection through cultures, complete documentation of surgical site cultures and antibiograms, and provision of informed consent.

Exclusion criteria

The exclusion criteria included open fractures, history of immunosuppression (patients on steroids and immunosuppressive drugs), incomplete medical documentation, and patient nonconsent.

Study measures

One thousand two hundred eighty-two patients were enrolled in the study, and all participants were monitored for one month to observe the development of SSIs during clinical follow-up. Samples were collected aseptically from infected surgical sites in brain heart infusion broth. Samples were transported within two hours to the Department of Microbiology. Culture was performed on 5% sheep blood agar and MacConkey agar. Identification of isolates was done using matrix-assisted laser desorption/ionization time-of-flight mass spectrometry, and antimicrobial susceptibility testing was carried out as per the latest Clinical and Laboratory Standards Institute guidelines. Data from all patients were recorded to facilitate comparisons between the infected and noninfected patients.

All patients were evaluated through detailed history taking and underwent a thorough general examination, along with systemic investigations that included a complete blood count, a random blood sugar test, kidney and liver function tests, and viral markers (HIV, hepatitis C virus, and hepatitis B surface antigen) to rule out any underlying infections. Demographic parameters, including name, age, sex, body mass index, and diagnosis, were recorded. Additionally, risk factors such as tobacco use, diabetes, hypertension, chronic renal or hepatic illnesses, previous drug intake history affecting surgical outcomes, duration of surgery, site of surgery, and type of surgery were noted.

Statistical analysis

The collected data were analyzed using Microsoft Excel 2018-19 (Microsoft Corporation, Redmond, WA) and GraphPad PRISM 5 software (GraphPad Software, San Diego, CA). Proportion, percentages, average, standard deviations (SDs), and p value were calculated for descriptive analysis.

## Results

Of the 1,282 enrolled subjects, only 98 (7.64%) developed SSIs; the remaining 1,184 were noninfected. Of the 1,282 patients, 812 (63%) were operated on in the elective operation theater, and the remaining 470 patients were treated in the emergency trauma center. Twenty-seven patients developed infections among elective cases, while 71 patients developed infections among emergency operated cases. The incidence was 2.21% in the elective cases and 5.53% in the emergency cases.

The mean age of the infected subjects, 39.57 ± 15.27 years, was found to be significantly higher (p < 0.0001) than that of the noninfected subjects (30.13 ± 10.11 years). Men were almost four times more likely to be infected than women. Of the total male subjects, 82 subjects (11.6%) were infected, whereas only 16 subjects (2.78%) of the total female subjects were infected. The most common infectious agent was *Staphylococcus aureus* in 23 subjects (22.54%), followed by *Escherichia coli* in 16 subjects (15.68%), *Klebsiella pneumoniae* in 14 subjects (13.72%), and *Acinetobacter baumannii* in 13 subjects (12.74%), accounting for approximately 65% ​​of all SSIs (Table 1).

**Table 1:** Bacteriological profile among infected patients

Infecting agent	Infected subjects (%)
Staphylococcus aureus	23 (22.54%)
Escherichia coli	16 (15.68%)
Klebsiella pneumoniae	14 (13.72%)
Acinetobacter baumannii	13 (12.74%)
Pseudomonas aeruginosa	8 (7.84%)
Proteus mirabilis	3 (2.94%)
Citrobacter werkmanii	2 (1.96%)
Staphylococcus epidermidis	2 (1.96%)
Enterococcus faecalis	2 (1.96%)
Acinetobacter lwoffii	1 (0.98%)
Sterile	14 (13.72%)
Total	98 (100%)

Diabetes and tobacco were the two most significant individual risk factors among the 98 infected individuals, with percentages of 26 subjects (26.53%) and 16 subjects (16.32%, respectively. Nearly 60% of the subjects were significantly affected by four key risk factors: diabetes, tobacco use, alcohol consumption, and smoking. Many subjects had two or more risk factors in common (Table 2).

**Table 2:** Risk factors among infected patients

S. no.	Risk factor	Number of subjects (%)
1.	Diabetes mellitus	26 (26.53%)
2.	Tobacco	16 (16.32%)
3.	Alcohol, tobacco	6 (6.12%)
4.	Hepatitis B	4 (4.08%)
5.	Hypertension	4 (4.08%)
6.	Smoking, tobacco	4 (4.08%)
7.	Alcohol	3 (3.06%)
8.	Smoking, alcohol	3 (3.06%)
9.	Diabetic, tobacco	2 (2.04%)
10.	Hemophilia	2 (2.04%)
11.	Alcohol, diabetes mellitus	1 (1.02%)
12.	Chronic kidney disease	1 (1.02%)
13.	Chronic myeloid leukemia	1 (1.02%)
14.	Multiple myeloma	1 (1.02%)
15.	Smoking	1 (1.02%)
16.	No risk factor	23 (23.47%)
Total		98 (100%)

Procedural risk factors, such as surgical time, surgery performed by junior surgeons, use of tourniquets, and emergency surgery, were significantly associated with SSI. The mean ± SD of surgery time for infected subjects was 130.7 ± 48.23 minutes, which was significantly longer than 112.0 ± 35.72 minutes for noninfected subjects. Of the 16 surgical sites studied, the femur was found to be infected more frequently. Hip, knee, and spinal surgeries followed this. The arthroscopy was completely free of infection (Table 3).

**Table 3:** List of involved surgical/anatomical sites ^*^Level of statistical significance, with a p value <0.0001

Surgical/anatomic site	Operated	Infected	% of infected/operated cases	Noninfected	Paired t-test p value between infected and noninfected cases
Total hip arthroplasty	149	13	8.72%	136	<0.0001^*^
Trauma: around knee	138	12	8.70%	126	
Trauma: around hip	131	11	8.40%	120	
Elective spine surgery	106	6	5.66%	100	
Emergency spine surgery	91	8	8.79%	83	
Arthroscopy	88	0	0%	88	
Acetabulum surgery	81	8	9.88%	73	
Total knee arthroplasty	77	5	6.50%	72	
Trauma: tibia	77	6	7.79%	71	
Trauma: forearm	72	5	6.94%	67	
Trauma: humerus	63	4	6.35%	59	
Trauma: limb reconstruction	58	5	8.62%	53	
Trauma: femur	55	9	16.36%	46	
Trauma: ankle	42	3	7.14%	39	
Trauma: shoulder	34	2	5.88%	32	
Trauma: hand	20	1	5%	19	
Total	1,282	98	-	1,184	

Among the most commonly used antibiotics for susceptibility testing, aztreonam and imipenem showed the best sensitivity/resistance ratios (S/R ratios) of 3.9 and 3.29, respectively. In contrast, meropenem, tetracycline, gentamicin, piperacillin-tazobactam, and ertapenem showed S/R values ​​between 1.18 and 2.44 (Table 4).

**Table 4:** Results of antibiotic sensitivity (susceptibility) test

Antibiotic class	Antibiotic	Staphylococcus aureus	Klebsiella pneumoniae	Escherichia coli	Acinetobacter baumannii	Pseudomonas aeruginosa	Proteus mirabilis	Staphylococcus epidermidis	Citrobacter werkmanii	Enterococcus	Acinetobacter lwoffii
Cephalosporin	Cefepime	17% sensitive	8.33% sensitive	62.5% sensitive	30.76% sensitive	50% sensitive	66% sensitive	-	100% resistance	-	50%sensitive
	Cefoxitin	30.43% sensitive	10% sensitive	50% sensitive	Not tested	Not tested	66% sensitive	100% sensitive	100% resistance	-	-
	Ceftriaxone	4.34% sensitive	33.33% sensitive	37.5% sensitive	11.11% sensitive	100% resistance	33.3% sensitive	-	100% resistance	-	-
	Ceftazidime	39.13% sensitive	11.12% sensitive	37.5% sensitive	15.38% sensitive	75% sensitive	33.3% sensitive	-	100% resistance	-	100%sensitive
	Cefazolin	16.6% sensitive	10% sensitive	50% sensitive	Not tested	Not tested	60% sensitive	-	100% resistance	-	-
	Cefoperazone sulbactam	32.4% sensitive	80% sensitive	50% sensitive	sensitive	100% sensitive	100% sensitive	-	100% resistance	-	-
Carbapenems	Meropenem	80% sensitive	85% sensitive	100% sensitive	20% sensitive	87.5% sensitive	100% sensitive	-	100% resistance	-	50% sensitive
	Ertapenem	21.73% sensitive	83.3% sensitive	43.75% sensitive	Not tested	Not tested	33% sensitive	-	100% resistance	-	-
	Imipenem	80% sensitive	90% sensitive	87.5% sensitive	15.38% sensitive	100% sensitive	100% sensitive	-	100% resistance	-	50% sensitive
Fluoroquinolones	Levofloxacin	15.38% sensitive	25% sensitive	43.75% sensitive	85% sensitive	60% sensitive	33% sensitive	100% sensitive	100% resistance	50% sensitive	50% sensitive
	Ciprofloxacin	18.75% sensitive	30% sensitive	45% sensitive	18.15% sensitive	37.5% sensitive	33% sensitive	100% resistance	100% resistance	50% sensitive	50% sensitive
Tetracycline	Minocycline	100% sensitive	100% resistance	6.25% sensitive	7.6% sensitive	Not tested	Not tested	-	-	-	50% sensitive
	Tetracycline	50% sensitive	28.57% sensitive	37.5% sensitive	53.84% sensitive	Not tested	33% sensitive	-	100% sensitive	70% sensitive	50% sensitive
Aminoglycosides	Amikacin	60% sensitive	57.14% sensitive	62.5% sensitive	23.03% sensitive	75% sensitive	66% sensitive		100% resistance	50% sensitive	100% sensitive
	Gentamycin	30.43% sensitive	50% sensitive	43.75% sensitive	30.76% sensitive	87.5% sensitive	66% sensitive	100% sensitive	100% resistance	50% sensitive	50% sensitive
	Tobramycin	50% sensitive	50% sensitive	37.5% sensitive	15.38% sensitive	37.5% sensitive	66% sensitive		100% resistance	-	-
Monobactam	Aztreonam	80% sensitive	57.14% sensitive	62.5% sensitive	Not tested	87.5% sensitive	100% sensitive		100% sensitive	-	-
Lincomycin	Clindamycin	80% sensitive	Not tested	Not tested	Not tested	Not tested	Not tested	-	-	-	-
Beta lactamase	Ampicillin	100% resistance	28.57% sensitive	50% sensitive	100% resistance	100% resistance	33.33%sensitive	-	100% resistance	-	-
	Amoxicillin	13.04% sensitive	22.22% sensitive	50% sensitive	Not tested	12.5% sensitive	66.6% sensitive	-	100% resistance	-	-
	Piperacillin and tazobactum	30% sensitive	85.7% sensitive	50% sensitive	33.23% sensitive	100% sensitive	100% sensitive	-	100% resistance	-	50% sensitive
	Penicillin	100% resistance	Not tested	100% resistance	Not tested	100% resistance	Not tested	100% resistance	Not tested	-	-
Sulfonamides	Cotrimoxazole	55.55% sensitive	28.57% sensitive	43.75% sensitive	30.76% sensitive	Not tested	70% sensitive	100% resistance	-	-	50% sensitive
Macrolides	Erythromycin	28.57% sensitive	Not tested	Not tested	Not tested	Not tested	Not tested	100% resistance	-	33% sensitive	-
Glycopeptide	Vancomycin	100% sensitive	Not tested	100% sensitive	100% sensitive	Not tested	100% sensitive	-	-	100% sensitive	-
	Teicoplanin	Not tested	Not tested	Not tested	50% sensitive	Not tested	Not tested	-	-	100% sensitive	-
Polypeptide	Polymyxin B	100% resistance	Not tested	Not tested	Not tested	Not tested	Not tested	-	-	-	-
	Colistin	100% sensitive	100% sensitive	100% sensitive	100% sensitive	Not tested	Not tested	-	-	-	-

## Discussion

Orthopedic surgical implants, including screws, plates, and joint prostheses, can create an environment that is highly conducive to the proliferation of infectious agents. The introduction of these foreign materials disrupts normal tissue integrity and impairs the body's immune response, making patients vulnerable to infections [15]. Consequently, postoperative SSIs have emerged as a critical challenge in orthopedic surgery. These infections not only prolong hospital stays and significantly increase medical costs but can also lead to severe complications, including the need for additional surgeries and the potential loss of functionality. Therefore, it is imperative to understand the associated risk factors and implement robust infection prevention strategies to safeguard patient health and enhance the outcomes of orthopedic procedures.

Advanced age is associated with a higher incidence of SSIs [16,17]. Taherpour et al. [18] reported that the median age of patients with orthopedic SSIs was 44 years. In contrast, Al-Mulhim et al. [19] found that the mean age of orthopedic SSI patients was 38.13 ± 19.1 years. Our study revealed a mean age of 39.57 ± 15.27 years for infected cases, which was significantly higher than the mean age of 30.13 ± 10.11 years for noninfected cases. Factors indirectly associated with age include a higher prevalence of comorbid conditions and weakened immune response to bacterial infections in older patients.

Several studies have indicated that men are more susceptible to SSIs than women, with an incidence of over 60% in men [19,20]. The present study observed a similar trend, with 83.67% of men affected compared to 16.33% of women.

In most studies, *S. aureus* has been identified as the most frequent cause of SSIs, with an incidence exceeding 25%. In some studies, this figure surpassed 50% [19-25]. However, some researchers have reported *E. coli* as the most common bacterium responsible for SSI [26-28]. Other significant pathogens identified in various studies include Pseudomonas, Klebsiella, Enterococci, and Acinetobacter species [29]. In the current study, *S. aureus* was also found to be the primary agent of SSI in orthopedic surgery, followed by *E. coli*. *K. pneumoniae*, *A. baumannii*, and *Pseudomonas aeruginosa* were the three common agents, accounting for over 30% of SSI cases.

This study identified several clinical and behavioral risk factors that were significantly associated with orthopedic SSIs. Diabetes was present in 26.5% of patients, while tobacco use affected 16.3%. Additional risk factors included a combination of alcohol and tobacco, smoking in conjunction with tobacco, smoking alongside alcohol, hypertension, and alcohol use alone, which were observed in 3%-6% of the subjects. Among other modifiable risk factors, a significant association was observed between surgical time, surgery performed by junior consultants, and SSI. A longer surgery duration means increased tissue exposure time to the external environment, which is possibly responsible for the increased infection rate [30].

We found that the odds of infection were more than 2.5 times higher in cases operated upon by junior surgeons than in those operated upon by senior surgeons. Surgery performed by junior consultants takes longer and has a higher infection rate. This is mainly because senior surgeons use refined techniques that minimize soft tissue dissection and damage, leading to better outcomes [31]. In our comparison of open and closed surgeries, we found that infections were four times more common in open surgery than in closed surgery. Closed surgery is a minimally invasive procedure involving arthroscopy, endoscopy, closed nailing, percutaneous plating, and screw insertion. When a tourniquet was not used, the risk of infection increased more than 1.5 times. Furthermore, we found that the use of tourniquets for greater than one hour increased the risk of infection. Not using it increases the risk of infection due to the accumulation of extra blood at the operation site. SSI was more than four times higher in emergency surgery than in elective surgery (15.11% and 3.33%, respectively).

In our study, the most sensitive antibiotics were vancomycin and colistin, with 100% sensitivity (although the total number of tests was insufficient to prove statistical significance). However, the antibiotics used in a substantial number of SSI samples with good S/R ratios were aztreonam, imipenem, meropenem, and amikacin, with S/R ratios of 3.9, 3.29, 2.44, and 1.96, respectively.

Limitations

Our study was conducted at a single center and focused on one department. The patient population consisted of individuals from various socioeconomic and ethnic backgrounds, making it difficult to compare populations in the developed Western world. We included patients who developed an infection within four weeks, but did not investigate the rate of late-onset infections. The duration of our study was one year, and we emphasized the need for a more extended study period to obtain more relevant results.

Strength and future scope

Our analysis highlights the need for a policy on antibiotic use to prevent infections. We identified the most common Gram-positive and Gram-negative bacteria in patients who developed SSIs and their associated risk factors. This information can assist in targeting these bacteria more effectively and in developing guidelines for managing infections in other patients. We need to conduct a level 1 multicenter study to create standardized guidelines for antibiotic usage.

## Conclusions

This study identified the most common bacteria associated with SSIs, their incidence rates, and antibiotic sensitivity. This critical information is essential for the effective empirical treatment of SSIs following orthopedic surgery. Additionally, it allows the establishment of robust treatment protocols that can identify high-risk patients before surgery, ensuring that appropriate postoperative antibiotics are promptly administered.
